# Positron Emission Tomography-Determined Hyperemic Flow, Myocardial Flow Reserve, and Flow Gradient—Quo Vadis?

**DOI:** 10.3389/fcvm.2017.00046

**Published:** 2017-07-17

**Authors:** Thorsten M. Leucker, Ines Valenta, Thomas Hellmut Schindler

**Affiliations:** ^1^Department of Medicine, Division of Cardiology, Johns Hopkins University School of Medicine, Baltimore, MD, United States; ^2^Department of Radiology, School of Medicine, Division of Nuclear Medicine, Johns Hopkins University School of Medicine, Baltimore, MD, United States

**Keywords:** CAD, myocardial ischemia, myocardial blood flow, myocardial flow reserve, multivessel disease, positron emission tomography, left ventricular wall motion

## Abstract

Positron emission tomography/computed tomography (PET/CT) applied with positron-emitting flow tracers such as ^13^N-ammonia and ^82^Rubidium enables the quantification of both myocardial perfusion and myocardial blood flow (MBF) in milliliters per gram per minute for coronary artery disease (CAD) detection and characterization. The detection of a regional myocardial perfusion defect during vasomotor stress commonly identifies the culprit lesion or most severe epicardial narrowing, whereas adding regional hyperemic MBFs, myocardial flow reserve (MFR), and/or longitudinal flow decrease may also signify less severe but flow-limiting stenosis in multivessel CAD. The addition of regional hyperemic flow parameters, therefore, may afford a comprehensive identification and characterization of flow-limiting effects of multivessel CAD. The non-specific origin of decreases in hyperemic MBFs and MFR, however, prompts an evaluation and interpretation of regional flow in the appropriate context with the presence of obstructive CAD. Conversely, initial results of the assessment of a longitudinal hyperemic flow gradient suggest this novel flow parameter to be specifically related to increases in CAD caused epicardial resistance. The concurrent assessment of myocardial perfusion and several hyperemic flow parameters with PET/CT may indeed open novel avenues of precision medicine to guide coronary revascularization procedures that may potentially lead to a further improvement in cardiovascular outcomes in CAD patients.

## Introduction

Positron emission tomography/computed tomography (PET/CT)-guided assessment of myocardial perfusion with concurrent quantification of myocardial blood flow (MBF) in milliliters per gram per minute by means of radiotracer kinetic modeling affords a comprehensive identification and delineation of subclinical and clinically manifest coronary atherosclerosis (1–6). Previous studies have suggested that a reduction in hyperemic MBFs and myocardial flow reserve (MFR = hyperemic MBF/rest MBF) improves prognostication to standard myocardial perfusion assessment (7–12). In asymptomatic patients with established cardiovascular risk factors, a dysfunction of the coronary circulation, as determined with PET flow measurements, is widely recognized as early functional stage of developing structural CAD (1, 5, 6, 13). Improvement or even normalization of hyperemic MBFs and MFR by primary preventive medical treatment, e.g., angiotensin-converting enzyme inhibitors or angiotensin II receptor blockers (14, 15), beta-hydroxymethylglutaryl coenzyme A reductase inhibitors (16), hormone replacement therapy in postmenopausal women (17), insulin-sensitizing thiazolodinedione in insulin-resistant individuals (18), glycemic control in diabetes (19), physical exercise (20), or gastric bypass-induced weight loss (21–23), have been put forth as an emerging therapeutic strategy for individualizing the primary preventive medical treatment of the CAD process and thereby improving cardiovascular outcomes (1, 4, 5). In respect of the diagnostic value of PET-determined flows, the focus has shifted toward the clinical application of hyperemic MBFs and MFR in patients with left main and/or advanced multivessel CAD (6). The addition of PET/CT-determined hyperemic MBFs and MFR to the conventional assessment of myocardial perfusion provides incremental diagnostic information to conventional scintigraphic myocardial perfusion imaging (MPI). For example, in patients with established CAD, the unraveling of a regional myocardial perfusion defect during pharmacologically stimulated hyperemic flows signifies the most severe epicardial narrowing, while less severe but flow-limiting stenosis in multivessel CAD can be identified through reductions in hyperemic flow, MFR, and the longitudinal flow gradient (1, 5, 6, 24–26). Such a diagnostic approach, incorporating several hyperemic flow parameters into the evaluation, may enable a comprehensive detection of downstream effects of “each” CAD lesion on hyperemic flow in multivessel disease. Nevertheless, reductions in hyperemic MBFs may not only result from advanced flow-limiting CAD lesions but also from a dysfunction of the coronary arteriolar vessels, or both, which can manifest in a relatively low specificity of the hyperemic MBF in the detection of multivessel CAD (3, 25, 27, 28). The interpretation of hyperemic MBFs and/or MFR in patients with multivessel CAD, therefore, calls for an appropriate synthesis of all available information of coronary morphology, microvascular function, and wall motion analysis in the diagnostic decision-making process (1, 6). The aim of this article is to discuss the potential role of the combined assessment of myocardial perfusion and MBF by PET/CT in the identification and delineation of multivessel CAD in the clinical routine.

## Methodology

Like for cardiac single-photon emission computed tomography (SPECT), PET assesses visually or semiquantitatively the relative distribution of the radiotracer uptake from the last static frame (e.g., 900 s) on stress and rest images of the left ventricular myocardium for the identification of regional myocardial ischemia (29). In addition, cardiac PET imaging with short-lived positron-emitting flow tracers injected intravenously, like ^13^N-ammonia, ^82^Rubidium, or ^15^O-water, and dynamic image acquisition of the radiotracer traversing through the pulmonary arterial system to its extraction and retention in the left ventricle (LV) affords the quantification of regional MBF in milliliter per gram per minute during vasomotor stress, at rest, and its MFR (MFR = hyperemic MBF/rest MBF). For the calculation of the MBF, one to three compartment models are available to characterize the first pass extraction and retention of the flow radiotracer in the myocardium. In addition, operational equations are used in order to compensate for physical decay of the radiotracer, partial volume-caused underestimation of the true myocardial tissue concentration (given a homogenous myocardial wall thickness of 10 mm), and spillover of radioactivity between right- and left ventricular blood pool and the myocardium. Such a non-invasive approach with PET to quantify MBF in milliliters per gram per minute has been validated for ^13^N-ammonia, ^82^Rubidium, or ^15^O-water against independent microsphere blood flow assessment at rest and during pharmacologically induced hyperemia (30–35).

## Relationship Between Stenosis and Coronary Flows

**Figure 1:**
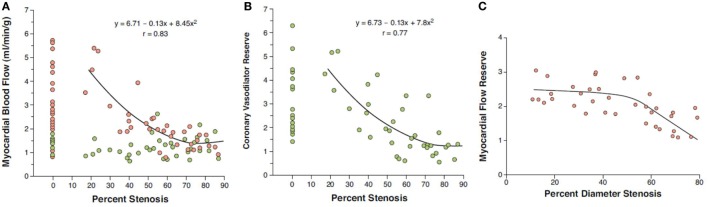
Coronary flow in relation to epicardial artery diameter stenosis (%). **(A)** At rest, no relationship between myocardial blood flow (MBF) and percentage coronary artery stenosis is noted (green circles), while there is an inverse relationship between hyperemic MBFs and percentage of focal epicardial narrowing during pharmacologic vasodilation (red circles). **(B)** Likewise, myocardial flow reserve (MFR = hyperemic MBF/resting MBF) demonstrates a comparable inverse relationship with percentage coronary artery stenosis (41). Yet, when analyzing stenoses of intermediate severity (40–70% diameter stenosis), a relatively high variability in individual MFR is realized. Of note, diminished hyperemic MBF or MFR in individuals without epicardial coronary artery stenoses may be comparable to those in myocardial regions subtended to epicardial lesions ≥50% diameter stenosis. **(C)** MFR commonly decreases when percent diameter stenosis exceeds ≥50% as measured with quantitative coronary angiography (correlation coefficient *r* = 0.77, root mean square error = 0.37, *p* < 0.00001) (41) [reproduced with kind permission from Schindler et al. (1)].

**Figure 2:**
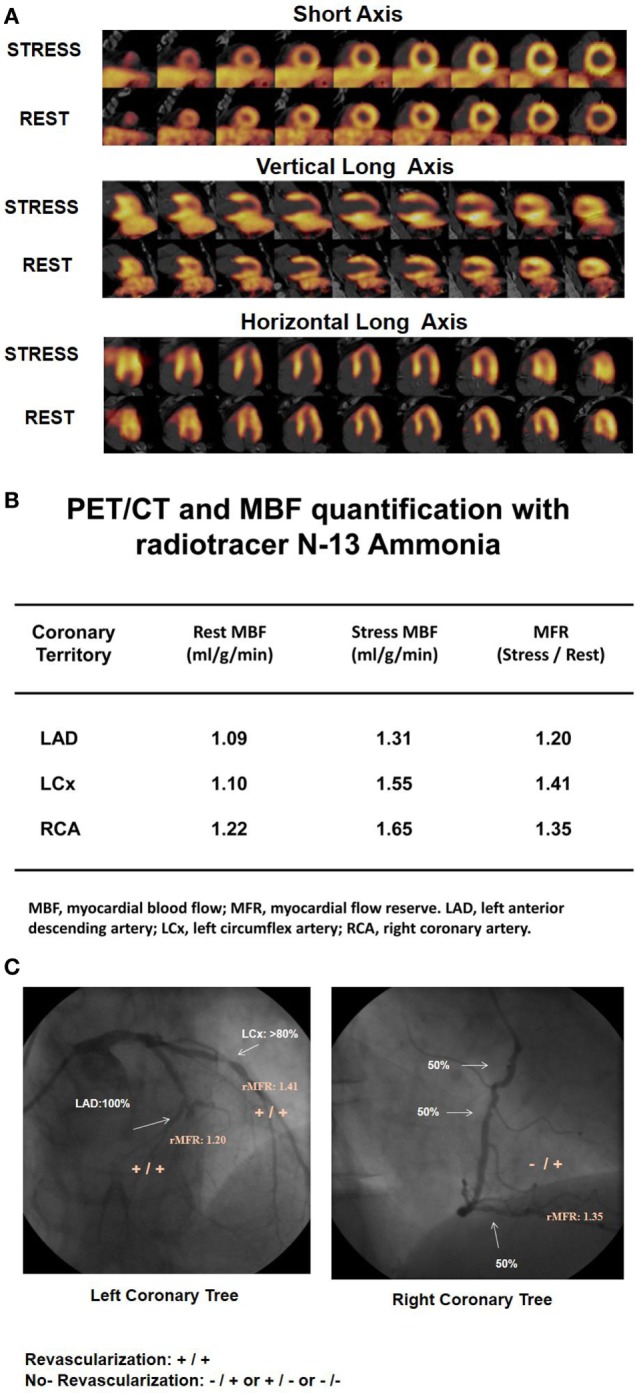
^13^N-ammonia positron emission tomography/computed tomography (PET/CT)-determined perfusion and myocardial blood flow (MBF) in multivessel CAD. A 61-year-old patient with arterial hypertension and type 2 diabetes mellitus presented with progressive shortness of breath and atypical chest pain. **(A)** On stress ^13^N-ammonia perfusion images, there is a moderate decrease in radiotracer uptake of the mid-to-distal anterior, anteroseptal, and apical regions of the left ventricle, which becomes reversible on the rest images to signify ischemia in the left anterior descending artery (LAD) distribution. ^13^N-ammonia uptake in the left circumflex artery (LCx) and right coronary artery (RCA) distribution is widely maintained. **(B)** Quantification of MBFs demonstrates globally reduced myocardial flow reserve (MFR) with a regional MFR of 1.20 in the LAD-, 1.41 in the LCx-, and 1.35 in the RCA distribution, respectively. **(C)** Invasive coronary angiography signifies three vessel disease with proximal occlusion of the LAD, 80% stenosis in the proximal segments of the LCX (left panel), and sequential 50–60% lesions in the RCA (right panel). When applying morphological and functional criteria with epicardial stenosis >70% and MFR <1.7 *(*criteria: +*/*+), then apart from the proximal LAD occlusion, the LCx lesion of less and intermediate severity (≈80%) is also identified as likely hemodynamic significant despite normal radiotracer uptake. Regarding the RCA, only one of the criteria apply. While regional MFR is markedly reduced with 1.35, the serial lesions of 50% do not reach the threshold of >70% diameter stenosis (criteria: −/+). Consequently, the distinct reduction in MFR in the RCA distribution may predominantly reflect microvascular dysfunction and not hemodynamically obstructive CAD [Reproduced with kind permission from Schindler et al. (1) and Schindler (6)].

**Figure 3:**
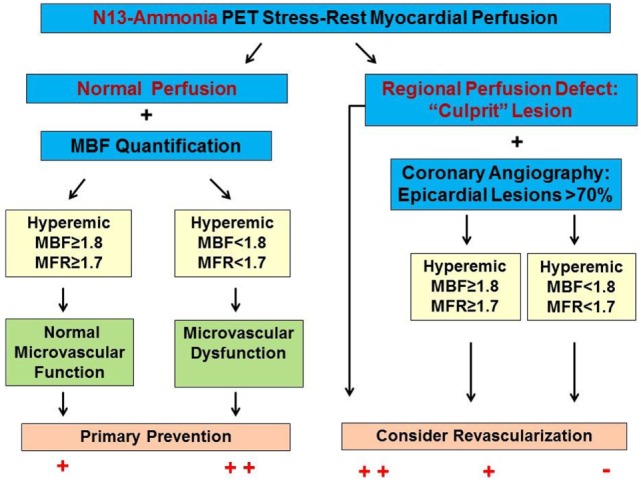
Algorithm for the Integration of ^13^N-ammonia positron emission tomography/computed tomography perfusion images and myocardial blood flows (MBFs) in multivessel CAD. In individuals with normal stress–rest myocardial perfusion images, the quantification of hyperemic MBF and myocardial flow reserve (MFR) may unravel microvascular dysfunction as functional precursor of CAD that may reinforce lifestyle-changes and/or preventive medical care. A stress-induced regional perfusion defect, however, indicates the most advanced CAD lesion. In this respect, adding hyperemic MBF and MFR may signify flow-limiting effects of lesions >70% diameter but less severe than observed for the culprit lesions and with normal radiotracer-uptake [reproduced with kind permission from Schindler (6)].

**Figure 4:**
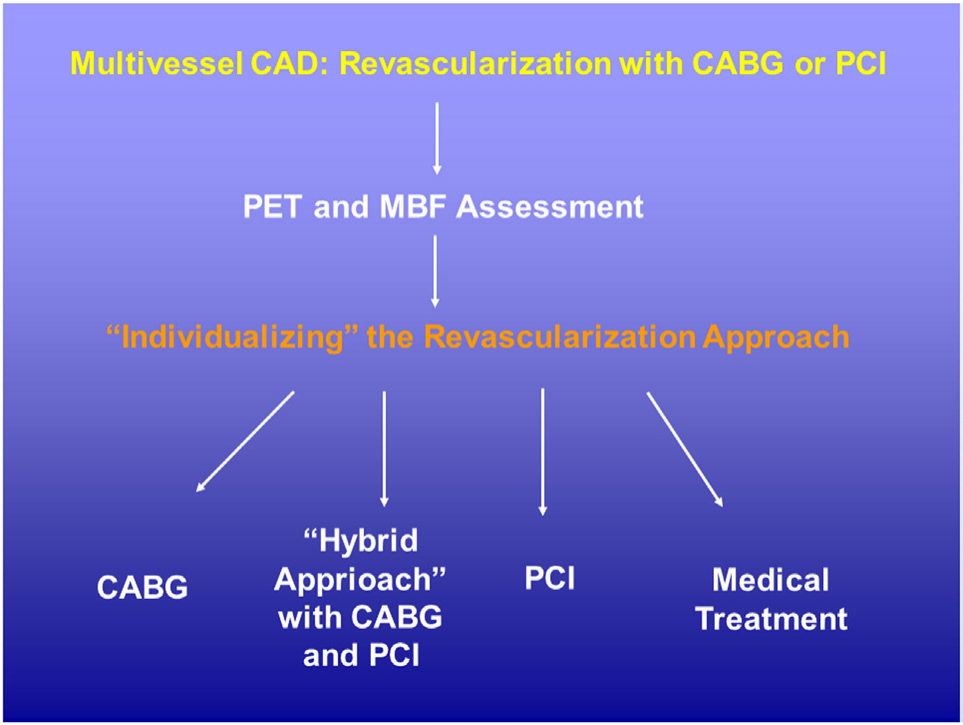
Potential role of positron emission tomography (PET) perfusion and flow quantification in patients with multivessel CAD disease. The evaluation of the hemodynamic significance of each single CAD lesions with the assessment of corresponding hyperemic MBF and/or MFR may aid in the decision-making process for coronary revascularization strategy. CABG, coronary aortic bypass graft; MBF, myocardial blood flow; MFR, myocardial flow reserve; PCI, percutaneous coronary intervention.

**Figure 5:**
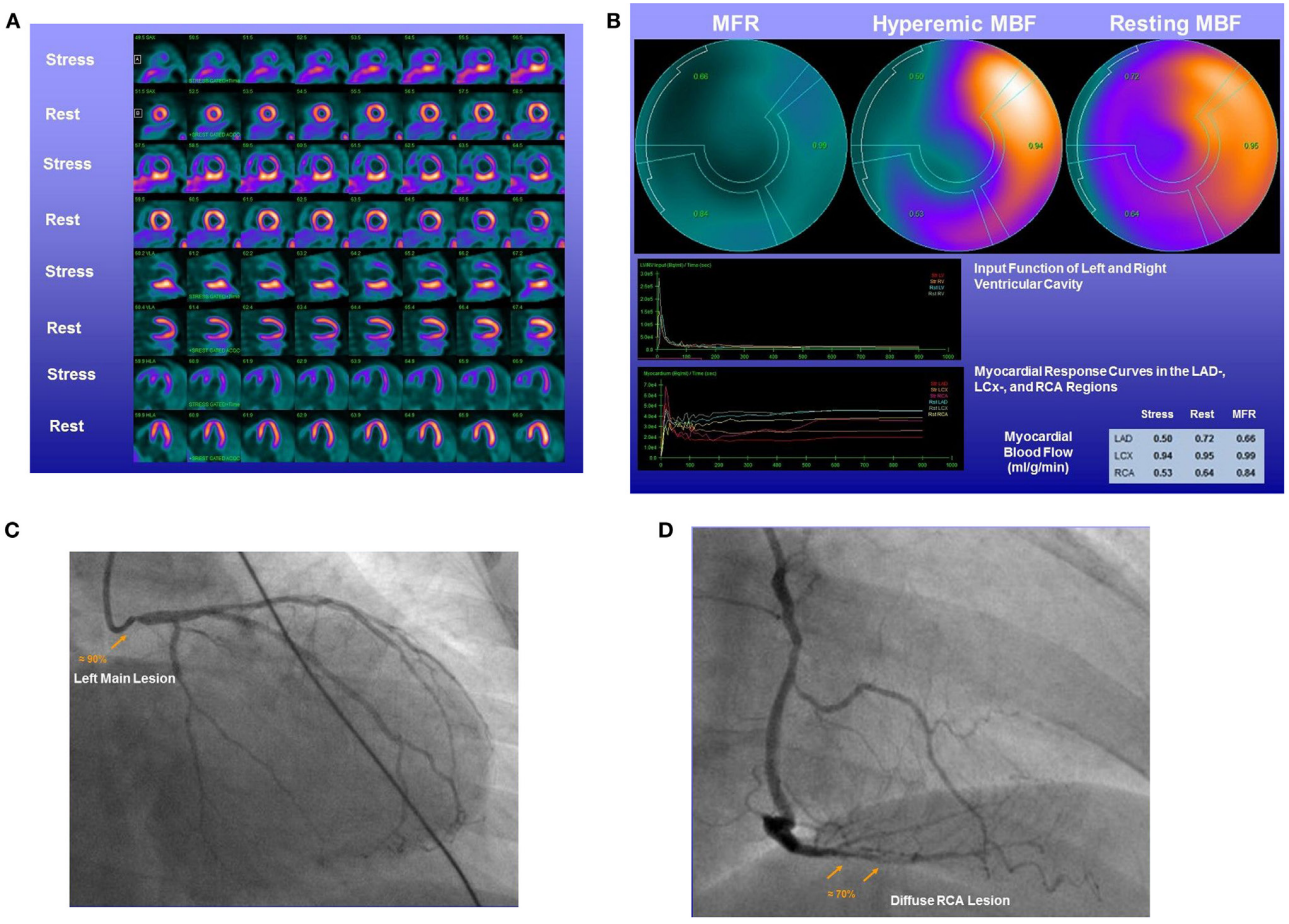
^13^N-ammonia positron emission tomography/computed tomography in the assessment of complex CAD. A 58-year-old women with type 2 diabetes mellitus and dyslipidemia presented with effort-induced angina symptoms. **(A)** On stress ^13^N-ammonia perfusion images, there is a severe decrease in radiotracer uptake of the anterior, anteroseptal, apical, and less severe in the anterolateral regions of the left ventricle, which becomes reversible on the rest images to signify large-sized, severe ischemia. On stress images, the inferior wall demonstrates pronounced radiotracer uptake likely due to overlapping high liver uptake that hampers an accurate evaluation. **(B)** Quantification of myocardial blood flows (MBFs) demonstrates a decrease of hyperemic MBFs from rest in all three major coronary artery territories of the left anterior descending artery (LAD)-, left circumflex artery (LCx)-, and right coronary artery (RCA) distribution. This resulted in regionally reduced myocardial flow reserves (MFRs) of 0.66 in the LAD-, 0.99 in the LCx-, and 0.84 in the RCA distribution, respectively. **(C)** Invasive coronary angiography signifies a high-grade lesion of the left main stem. **(D)** In addition, a diffuse ≈70% lesions in the distal RCA are noted. Consequently, the distinct reduction in MFR in the RCA distribution also unmasks the hemodynamic significance of the mid RCA lesions, apart from the left main, and the patient was referred for surgical revascularization of both arteries.

Numerous PET flow studies have demonstrated that hyperemic MBFs and MFR commonly start to decrease when a lesion exceeded 50% of luminal diameter (Figures 1A–C) (25, 26, 36–43). Conversely, a substantial variability of the individual hyperemic MBFs and MFR is observed and is commonly related to a different degree of increases in vasodilatory capacity of the coronary microcirculation to balance CAD-related focal increases in epicardial resistance and/or the presence of collateral flow (44–46). Findings of the “clinical outcomes utilizing revascularization and aggressive drug evaluation” (COURAGE) trial put forth that physical exercise and/or preventive medical intervention may contribute to avoid the manifestation of stress-related myocardial ischemia or reduce the ischemic burden by improving the vasodilator capacity of the coronary circulation and thus increasing hyperemic MBFs and MFR, respectively (47). In addition, hypoxia-induced collateral flow is also likely to have counterbalanced the manifestation of stress-induced myocardial ischemia in stable CAD patients in the COURAGE cohort (47). Thus, it may not be surprising that despite an epicardial narrowing of ≥50%, stress-induced regional myocardial ischemia may be encountered in only 30% of such patients (48, 49). It is also important to bear in mind that hyperemic MBFs may be substantially diminished owing to adverse effects of cardiovascular risk factors induced increases in oxidative stress burden and inflammation within the coronary arteriolar wall even in the absence of obstructive CAD (50–52). Reductions in hyperemic MBFs and MFRs *per se*, therefore, cannot be differentiated between the presence of flow-limiting, obstructive CAD lesions, and cardiovascular risk factors-induced microvascular dysfunction. This fact explains the reported relative low specificity of hyperemic flows in CAD detection (25–27). Nevertheless, when the severity of an epicardial narrowing increase, the coronary resistance shifts from the coronary arteriolar vessels to the level of the epicardial conductance vessel as the compensatory increase in vasodilatory capacity of the microcirculation becomes depleted (36–38, 53). The evaluation of an impairment in hyperemic MBFs and MFR in conjunction with coronary morphology, therefore, is of utmost importance for an adequate evaluation and clinical decision-making in patients with multivessel CAD (1, 6). In this direction, Gould et al. (2) have put forth that for a CAD narrowing exceeding 70%, decreases in MFR <1.7 can be deemed to be widely related to stenosis-induced epicardial resistance to hyperemic MBF. Adding information about the coronary morphology to PET-determined hyperemic MBFs and/or MFR, therefore, may overcome the non-specificity of regional hyperemic flow increases (1). Consequently, the detection of a regional myocardial perfusion defect during pharmacologically stimulated hyperemic flows demonstrates the most severe epicardial narrowing, whereas an impairment of the MFR of less than 1.7 identifies downstream, flow-limiting effects of lesions on regional hyperemic flows even when no regional perfusion defect is apparent (Figures 2A–C) (6). Several investigations have compared the post-stenotic coronary flow velocity reserve with corresponding regional myocardial perfusion deficit on SPECT images and are in support of the use of the MFR for the identification of the hemodynamic significance of CAD lesions (Figure 3) (6). The optimal threshold for PET/CT-determined hyperemic MBFs or MFR to identify the hemodynamic effects of coronary artery lesions, however, is dependent on PET methodology, various positron-emitting radiotracers used for MBF quantification, and different reference standards such as coronary anatomy with stenosis ≥50 or ≥70%, invasively determined fractional flow reserve (FFR), or regional ischemia (1, 4, 6, 54, 55). For example, applying PET/CT and the blood flow radiotracer ^13^N-ammonia, the diagnostic value of hyperemic MBF, MFR, and the relative radiotracer content (millicuries per milliliter) for detecting ≥70% epicardial lesions was the highest, when a hyperemic MBF threshold value of ≥1.85 ml/g/min was used (56). Notably, the diagnostic accuracy for CAD detection with ^13^N-ammonia PET/CT demonstrated highest value of 0.90 for adenosine-stimulated absolute hyperemic MBF, followed by 0.86 for MFR, and 0.69 for ^13^N-ammonia relative uptake. Thus, when applying the threshold of ≈1.85 ml/g/min for hyperemic MBFs as determined with ^13^N-ammonia PET/CT, hyperemic MBFs appear to outperform the MFR as well as conventional myocardial perfusion scintigraphy in CAD detection (56, 57). Additionally, previous invasive investigations with intracoronary Doppler flow measurements of flow velocities commonly have defined 2.0 as optimal threshold for the MFR in differentiating between normal and abnormal hyperemic flow increases (58–60). The latter threshold of MFR of 2.0 derived from invasive flow studies has been translated to PET/CT perfusion imaging with ^13^N-ammonia or ^82^Rubidium as flow radiotracer (5). For ^82^Rubidium PET flow measurements, Johnson and Gould (54) identified an optimal MFR threshold of 1.74 with an AUC = 0.91 for pharmacologically induced regional perfusion deficit with severe angina and/or significant ST-segment depression. ^15^O-water is another positron-emitting flow tracer that is increasingly applied for PET/CT myocardial flow studies in a few centers in Europe. For ^15^O-water, the optimal thresholds for the detection of flow-limiting epicardial lesions, as defined by epicardial stenosis >90% and/or invasively determined FFR ≤0.80, have been reported for hyperemic MBF with 2.3 ml/g/min and for MFR with 2.50, respectively (61, 62). Defining such thresholds in hyperemic MBFs and MFR for different PET flow radiotracers enables the assessment of the hemodynamic significance of each CAD lesion (2) that may assist in the evaluation and clinical decision-making to gear coronary revascularization options with percutaneous transluminal coronary angioplasty, coronary artery bypass grafting, or hybrid interventions in individuals with complex and advanced multivessel disease (Figures 4 and 5). Although it remains to be clinically tested if this novel concept of an individualized coronary revascularization strategy with the help of PET-measured hyperemic flow increases may also translate into an improved cardiovascular outcome as compared to standard coronary surgical revascularization in patients with multivessel CAD.

## Longitudinal Flow Decrease

**Figure 6:**
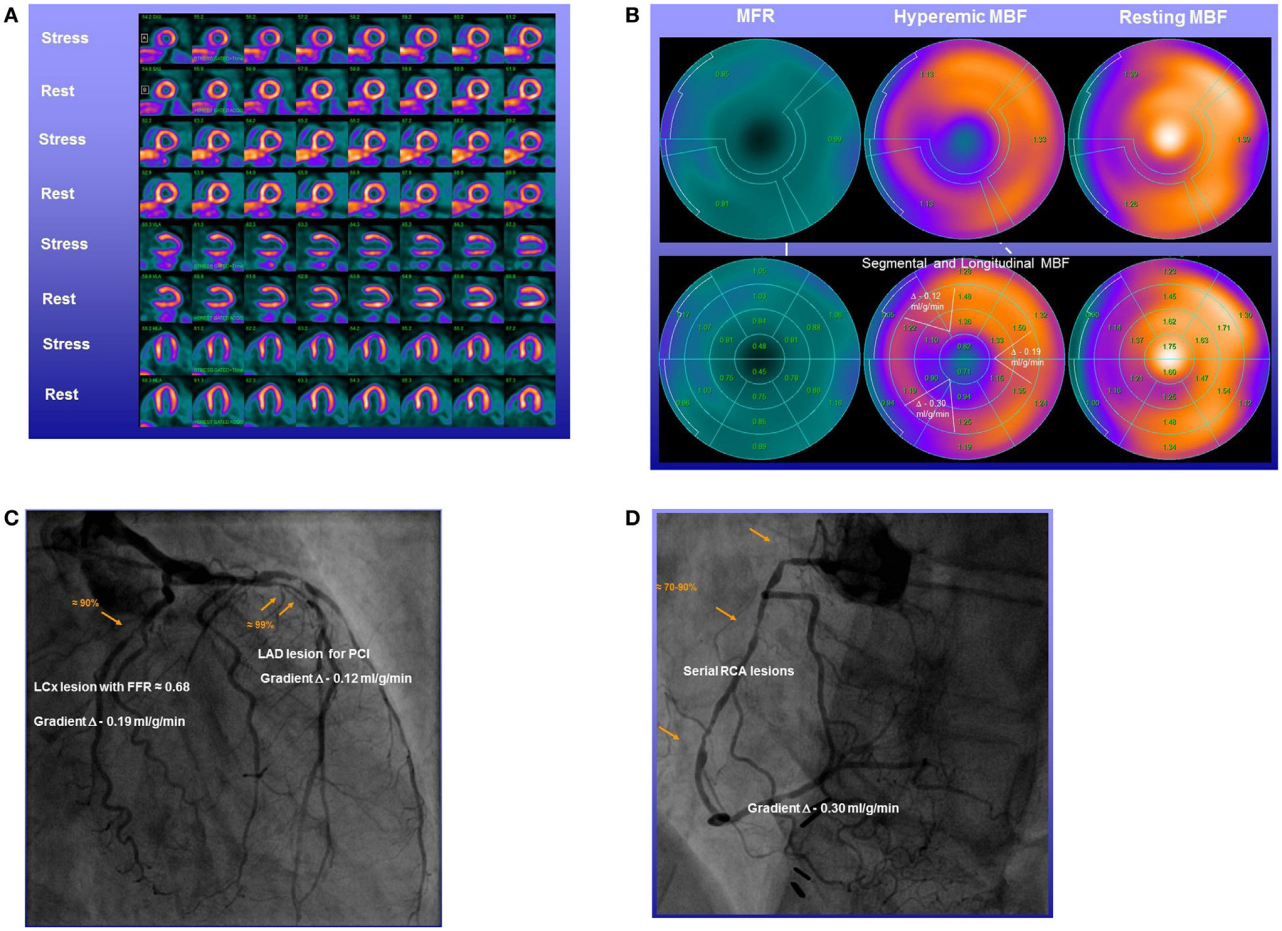
Abnormal longitudinal flow gradient in multivessel CAD. Stress–rest ^13^N-ammonia positron emission tomography/computed tomography (PET/CT) myocardial perfusion and flow quantification with ^13^N-ammonia PET/CT in a 58-year-old man with effort-induced angina chest pain. **(A)** Regadenoson-stress and rest ^13^N-ammonia PET/CT images in corresponding short-axis (top), vertical long-axis (middle), and horizontal long-axis (bottom) slices. On stress images, a moderate-to-severe decrease in myocardial perfusion, involving the antero-septo-apical and apical wall, is observed that normalizes on rest images to indicate ischemia in the left anterior descending artery (LAD) distribution. **(B)** Regional myocardial blood flow (MBF) quantification demonstrates abnormally reduced hyperemic MBFs and myocardial flow reserve (MFR) in all three major coronary artery territories of the LAD, left circumflex artery (LCx), and right coronary artery (RCA), respectively (upper panel). Segment MBF analysis unmasks a decrease in MBF from the mid to distal segments with a mean longitudinal MBF gradient during hyperemic flow in the LAD (0.12 ml/g/min), LCx (0.19 ml/g/min), and RCA (0.30 ml/g/min) (lower-middle panel). **(C)** Invasive coronary angiography of the left coronary artery in this patient showed a long 99% stenosis in the mid-LAD that was responsible for the observed stress-induced perfusion defect of the antero-septo-apical and apical walls on ^13^N-ammonia PET/CT perfusion images. In addition, large caliber diagonal branches of the LAD present a ≈70–80 and ≈80–90% stenosis, respectively. The proximal LCx has a proximal ≈80–90% stenosis. Invasively measured fractional flow reserve (FFR) of the proximal LCx lesion was abnormally reduced with 0.68 emphasizing also the hemodynamic significance of this lesion. **(D)** Invasive coronary angiography of the RCA demonstrates serial epicardial lesion from the proximal to mid segments of ≈70–90%, respectively [adapted with kind permission of Valenta et al. (26)].

**Figure 7:**
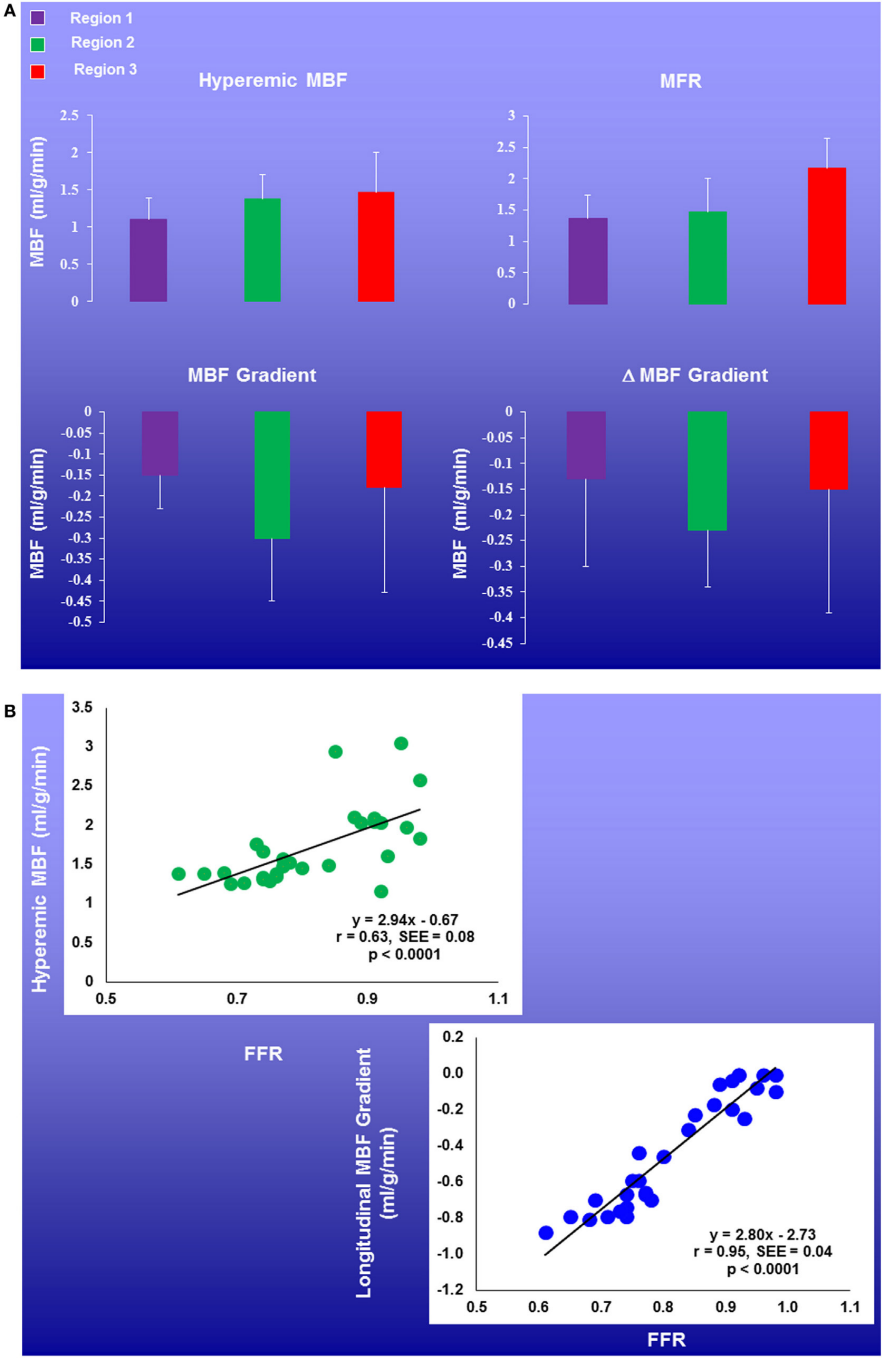
Regional myocardial blood flow (MBFs) and longitudinal MBF gradients in patients with multivessel CAD. Coronary flow parameters were assessed in the myocardial region with stress-related perfusion defect and with stenosis ≥50% (Region 1), without defect but with stenosis ≥50% (Region 2), or without stenosis ≥50% (Region 3). There was a mild and progressive increase in hyperemic MBFs and myocardial flow reserve (MFR) from region 1, region 2, to region 3. Further, the longitudinal MBF gradient during hyperemic flows was more pronounced in Region 2 than in Regions 1 and 3, respectively **(A)**. Of note, the longitudinal MBF gradient during hyperemic flows significantly correlated with the fractional flow reserve (FFR) (*r* = 0.95, *p* < 0.0001), while this association was less pronounced for corresponding MFR (*r* = 0.50, *p* = 0.006) **(B)** [adapted with kind permission of Valenta et al. (26)].

As an impairment of hyperemic MBFs and MFR in CAD may origin either from CAD-related epicardial narrowing, from microvascular dysfunction, or from both (1, 5, 63), reduced hyperemic flow and MFR are commonly seen as non-specific flow parameters. Some help may come from a more specific flow parameter such as a longitudinal decline in flow from the base to the apex of the heart or so-called hyperemic longitudinal MBF gradient to identify and characterize flow-limiting CAD lesions (24–26, 64–66). A longitudinal MBF gradient of the LV is assumed to be induced by CAD caused increases in epicardial resistance during hyperemic coronary flows (67–69). Pioneer investigations conducted by Gould et al. (70–74) were first to relate the longitudinal decrease in myocardial perfusion of flow to the presence of diffuse CAD. More recently, the reported longitudinal flow decrease or gradient during hyperemic flow increases was also related to stenosis severity and invasively measured FFR as functional parameter (25, 26) (Figure 6). The concept of the longitudinal MBF gradient is based on the Hagen–Poiseuille equation (53, 67, 68), which describes the dependency factors of intracoronary resistance such as a direct relation to flow velocity and inversely to the fourth power of the vessel diameter. In the normal coronary circulation, an increase in coronary flow and thus velocity due to a metabolic-mediated vasodilation in the microcirculation in response to an increase in oxygen demand induces a flow-mediated vasodilation of the epicardial artery that again reduces the velocity-induced increase in resistance in order to ascertain a low coronary resistance at the level of the epicardial conductance system (64, 75–77). The presence of diffuse CAD and/or advanced focal CAD lesion, however, will prevent or reduce an appropriate flow-induced and endothelium-dependent vasodilation of the epicardial artery and the additional intraluminal obstruction will cause an increase in epicardial resistance accompanied by a continuous decrease in intracoronary pressure from proximal-to-distal (68) that may manifest as longitudinal decrease in MBF or MBF gradient (25, 26, 66, 71, 78). Thus, the identification and characterization of a hyperemic longitudinal MBF gradient with PET/CT could indeed evolve to a unique and specific flow parameter to signify flow-limiting CAD lesions. For example, Valenta et al. (25) evaluated the diagnostic accuracy of the reported hyperemic longitudinal MBF gradient and the MFR for the detection of epicardial narrowing ≥50% in patients with prevalently multivessel disease CAD. Regional and segmental MBFs were determined and grouped in three territories. Territory 1 (T1): myocardial region with stress-induced perfusion defect and with stenosis ≥50%; territory 2 (T2): without defect but with stenosis ≥50%; or territory 3 (T3) without stenosis ≥50%. A hyperemic longitudinal MBF gradient was defined as a decline in segmental MBF from the mid to mid-distal left-ventricular myocardium. As it was observed, the Δ longitudinal MBF gradient (=longitudinal MBF gradient during hyperemia − longitudinal MBF gradient at rest) continuously rose from T1 to T2 and T3, respectively, when signified as median and interquartile range: −0.10 (−0.14, 0.03) and −0.21 (−0.35, −0.10) and −0.29 (−0.45, −0.14). There was a close association between the severity of epicardial artery stenosis and the longitudinal MBF gradient (*r* = 0.52, *p* < 0.0001) for the entire CAD study group with focal lesions ≥50% (T1 to T2), whereas such association was less pronounced for corresponding MFR (*r* = −0.40, *p* < 0.003) (25). Furthermore, when employing thresholds for the Δ longitudinal MBF gradient and MFR of ≤0.25 ml/g/min and ≤1.40, the diagnostic accuracy for the detection of coronary narrowing ≥50% was higher for the Δ longitudinal MBF gradient as compared to the conventional MFR (86 vs. 70%). Similarly, the sensitivity and specificity of the Δ longitudinal MBF gradient also proved to be significantly higher in comparison to corresponding MFR (88 vs. 71 and 81 vs. 63%, respectively). The combination of both flow parameters, however, rose the diagnostic accuracy and sensitivity to 94 and 100%, respectively, whereas the specificity turned intermediate to 75% (25). In a more recent investigation (26), the ^13^N-ammonia PET/CT-determined longitudinal MBF gradient and MFR were evaluated in direct comparison to invasively measured FFR in 29 patients with suspected or known CAD. Regional and segmental MBFs were determined as follows: region 1: stress-related perfusion defect and with stenosis ≥50%; region 2: without defect but with stenosis ≥50%; or region 3 without stenosis ≥50%. The PET-measured hyperemic longitudinal MBF gradient proved to be more severe in Region 2 than in Regions 1 and 3, respectively [median (IQR): 0.46 (20.70, 20.10) vs. 20.17 (20.29, 20.11) and 20.15 (20.25, 20.09) ml/g/min, respectively] (Figure 7A). Additionally, the hyperemic longitudinal MBF gradient significantly correlated with the FFR (*r* = 0.95, *p* < 0.0001). The observed correlation, however, was less prominent when the invasively determined FRR was related to corresponding MFR (*r* = 0.50, *p* = 0.006) (Figure 7B). Such observations further support the potential role of the hyperemic longitudinal MBF gradient as a novel and promising flow parameter to identify flow-limiting effects of CAD lesions in multivessel disease. However, despite these promising initial observations, there are still important limitations in methodology and hemodynamic parameters hampering the clinical application of the longitudinal MBF gradient in the detection and characterization of direct, downstream effect of focal CAD lesions on hyperemic coronary flows (25, 65, 79). Consequently, this novel approach in CAD detection and evaluation with the longitudinal MBF gradient may manifest with a certain variability and also underestimation, warranting further investigations. With the advent of MRI-PET (80–83), the apical myocardial segments may be less prone to partial volume effects, which may yield more accurate determination of the longitudinal MBF gradient. In addition, three-dimensional fusion of CT-determined coronary morphology and myocardial flow on a voxel basis (57) appears promising to render the longitudinal flow gradient assessment more effective (6).

## The Diagnostic Challenge: Diffuse Ischemia

**Figure 8:**
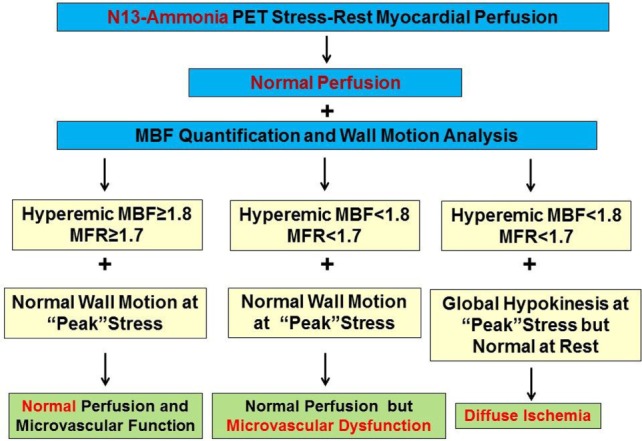
Algorithm for the integration of ^13^N-ammonia positron emission tomography/computed tomography perfusion images, myocardial blood flow (MBF), and wall motion analysis for differentiation between microvascular dysfunction and diffuse ischemia. Evaluating hyperemic MBFs in conjunction with wall motion analysis at “peak” stress affords the differentiation between predominant microvascular dysfunction and diffuse myocardial ischemia caused by significant left main and/or three vessel CAD. Balanced reductions in hyperemic MBFs and normal wall motion of the left ventricle (LV) at peak stress favors the presence of predominantly microvascular disease but not diffuse ischemia, whereas diffuse reductions in hyperemic MBFs associated with transient ischemic cavity dilation of the LV during vasomotor stress identifies the presence of diffuse ischemia [reproduced with kind permission from Schindler et al. (1)].

Conventional scintigraphic MPI evaluates the “relative” radiotracer uptake of the LV to identify regions with relative lower radiotracer uptake or perfusion deficit in comparison to the remaining myocardium. In multivessel disease, this scintigraphic imaging approach of myocardial perfusion may be limited as a relative decrease in regional radiotracer (perfusion deficit) may signify the most advanced CAD lesion, whereas the remaining remote regions subtended to less severe or stenosis of intermediate severity may still present a homogenous radiotracer uptake. Therefore, conventional stress–rest myocardial scintigraphy commonly detects clinically manifest multivessel CAD by unraveling stress-induced regional ischemia in the territory subtended to the most advanced or so called culprit lesions. Conversely, the remaining less severe but hemodynamically flow-limiting stenosis may be missed in such setting. Furthermore, diffuse ischemia induced by hemodynamically significant left main stenosis and/or advanced three vessel disease may also remain undetected by conventional myocardial scintigraphy. In such situations, no regional difference in radiotracer uptake on scintigraphic myocardial perfusion images may be observed, because diffuse ischemia of the LV creates “balanced” impairment of hyperemic MBFs (84). For example, in patients with left main disease (≥50% stenosis) and ≥70% stenosis of the right coronary artery or three vessel disease with ≥70% epicardial narrowing in each major vessel, only 10% (14/143) demonstrated some regional perfusion difference (85). When post-stress SPECT determined regional wall motion abnormalities were also included in the evaluation of stress–rest MPI, the detection of multivessel CAD rose but merely to 25% (85). Furthermore, Berman et al. previously evaluated the added diagnostic value of gated SPECT-determined global and regional wall motion of the LV in the detection of hemodynamically significant left main CAD (≥50% diameter stenosis) with exercise or adenosine stress ^99m^Technetium sestamibi SPECT MPI in 101 patients without prior myocardial infarction or coronary revascularization (86). In this investigation, a high-risk feature on myocardial perfusion images with moderate to severe regional ischemia (>10% myocardium at stress) was demonstrated in 59% of these patients. Yet, a combined analysis of abnormal perfusion and wall motion on post-stress-gated SPECT elevated the detection of high-risk cardiovascular individuals to 83% (86). Given the described limitation of conventional myocardial perfusion scintigraphy to accurately identify diffuse ischemia, the unique feature of PET/CT to determine not only global but also regional hyperemic MBF and MFR as well as wall motion analysis with gated PET/CT at peak stress may indeed overcome the limitations of conventional MPI. In principle, diffuse decreases in hyperemic MBFs and MFR may be related to the presence of diffuse ischemia (Figure 8). Yet, a diffuse impairment of left-ventricular hyperemic MBFs and/or MFR may originate from left-main and/or multivessel CAD, pronounced microvascular dysfunction, or both (1). In this direction, adding the wall motion analysis of gated PET at “peak” stress is critical in the interpretation of the perfusion images. As pronounced diffuse ischemia should lead to global myocardial stunning of the LV associated with a “peak” stress transient ischemic cavity dilation (TID) on gated PET images, the presence of TID at peak stress should be included to identify diffuse ischemia owing to advanced multivessel CAD (87, 88). If no TID is present, diffuse reductions of hyperemic MBFs and MFR may predominantly be related to microvascular dysfunction rather than to CAD-related increases in epicardial resistance (Figure 8). Interestingly, Naya et al. (27) reported that normal hyperemic MBFs carried a high negative predictive value of 97% in excluding high risk CAD as evidenced with invasive coronary angiography. Furthermore, the calculation of the LV ejection reserve (ΔLVEF = stress LVEF − rest LVEF) adds further important information for the exclusion of high risk CAD. In this respect, a LVEF reserve increase of >5% reflected a positive predictive value of only 41%, while the negative predictive value was as high as 97%. Thus, when PET analysis yields normal hyperemic MBFs in conjunction with normal to high LVEF reserve, high risk CAD can be widely excluded (27, 87). Taken together, the comprehensive analysis of hyperemic flows, left-ventricular wall motion at “peak” stress may enable to differentiate between diffuse ischemia and pronounced microvascular dysfunction. However, this concept needs be further confirmed in large-scale clinical investigations.

The latter described concept, however, may not necessarily apply in patients with ischemic cardiomyopathies and preexisting low left ventricular function. Ischemic preconditioning of the LV stimulates cardioprotective mechanisms striving to avoid further aggravation of left ventricular function (89, 90). Therefore, even in the presence of diffuse ischemia, only a minor or even no further decrease in global left ventricular function may ensue. Thus, the absence of a significant drop in LVEF during peak stress from baseline in patients with significant cardiomyopathies does not allow definite differentiation between diffuse ischemia and pronounced microvascular dysfunction. In addition, an impairment of hyperemic MBFs and MFR is a common finding in patients with both ischemic and non-ischemic cardiomyopathies (91). In such circumstances, non-invasive or invasive coronary angiography might be of added value to identify significant left-main and/or advanced multivessel CAD that otherwise could be missed by PET perfusion and flow quantification.

## FFR and MFR in Multivessel CAD

The FFR defines the fraction of maximal coronary flow passing through a narrowed artery segment. This is represented as a percentage of coronary flow through the same artery assuming the absence of a stenosis (67). Accordingly, the FFR, determined as the ratio of the absolute distal coronary pressure (after the lesions) and proximal pressure (before the lesion), is equal to the aortic pressures determined during adenosine-stimulated maximal hyperemia. In contrast to the MFR, the FFR is assumed to be widely independent of coronary driving pressure, heart rate, systemic blood pressure, and the status of the microcirculation (92, 93), and, therefore, provides specific information on epicardial lesion-related increase in resistance to hyperemic flow increases. An FFR ≥0.80 commonly signifies the absence of a hemodynamically significant stenosis. The application of invasively determined FFR has been proven clinically useful in the decision-making for coronary revascularization procedures in patients with multivessel CAD (94–96). For example, the multicenter FAME (Fractional Flow Reserve vs. Angiography for Multivessel Evaluation) trial (96) demonstrated that the application of FFR measurements in patients with multivessel CAD undergoing percutaneous coronary intervention (PCI) with drug-eluting stents significantly reduced composite end point of death, non-fatal myocardial infarction, and repeat revascularization as compared to angiography-guided PCI alone. The 1-year event rate proved to be significantly lower with 31.2% (67 patients) in the FFR group as compared to 18.3% (91 patients) in the angiography group (96). As outlined before (1, 6), conventional stress–rest scintigraphic MPI with proof of regional myocardial ischemia commonly signifies the most severe lesion in patients with multivessel disease, while less severe but hemodynamically significant lesions in other coronary distributions may be missed. In this respect, invasively determined FFR has been demonstrated to unravel up to 36% of ischemic regions otherwise missed by MPI in patients with multivessel disease (97). A substantial discordance up to one-third of cases between invasively measured FFR and PET-determined MFR has been reported (3, 62, 74). Adverse effects of cardiovascular risk factors such as arterial hypertension, hypercholesterolemia, smoking, and diabetes mellitus may lead to coronary microvascular dysfunction resulting in an impairment of hyperemic MBFs and MFR (1, 77). Pronounced microvascular dysfunction may in fact inhibit submaximal or maximal hyperemic flow increases during adenosine-stimulated vasodilator effects on the arteriolar vessels. Under such condition, the reduced hyperemic flow may not be sufficient to build up a flow or pressure gradient across a focal lesion and the FFR may remain normal (3). Conversely, an epicardial lesion may have an abnormal FFR <0.80, signifying a significant pressure decrease across the lesion, but still have normal hyperemic flow increase and MFR compensating for lesion-induced increase in epicardial resistance (2, 37, 38). In regards to coronary pathophysiology, it becomes obvious that FFR and MFR provide different information (2, 74). Given such, these flow parameters in fact may be additive to each other in different clinical settings (2, 3). Refinement and improved measures of segmental flow quantification to reliably determine a longitudinal MBF gradient during hyperemic flow stimulation (25, 26, 66) may provide a further step to a non-invasive FFR and comprehensive flow assessment with PET imaging.

## Conclusion

The concurrent assessment of myocardial perfusion and several hyperemic flow parameters with PET/CT may open new pathways of precision medicine to guide coronary revascularization procedures that may potentially lead to a further improvement in cardiovascular outcome in CAD patients. However, to solidify the clinical applicability of the above described concepts, further clinical investigations are needed.

## Author Contributions

TL wrote the initial draft of the article and obtained images. IV performed the literature search, obtained images, and edited the draft of the article. TS edited the manuscript and prepared the final figures.

## Conflict of Interest Statement

The authors declare that the research was conducted in the absence of any commercial or financial relationships that could be construed as a potential conflict of interest.

## Abbreviations

ARB, angiotensin II receptor blocker; CABG, coronary artery bypass grafting; CAD, coronary artery disease; FFR, fractional flow reserve; LAD, left anterior descending artery; LCx, left circumflex artery; LV, left ventricle; LVEF, left ventricular ejection fraction; MBF, myocardial blood flow; MFR, myocardial flow reserve; MPI, myocardial perfusion imaging; MRI, magnetic resonance imaging; PCI, percutaneous coronary intervention; PET/CT, positron emission tomography/computed tomography; PTCA, percutaneous transluminal coronary angioplasty; RCA, right coronary artery; SPECT, single-photon emission computed tomography; TID, transient ischemic cavity dilation.
